# Exploring the economic and social effects of care dependence in later life: protocol for the 10/66 research group INDEP study

**DOI:** 10.1186/2193-1801-3-379

**Published:** 2014-07-28

**Authors:** Rosie Mayston, Mariella Guerra, Yueqin Huang, Ana Luisa Sosa, Richard Uwakwe, Isaac Acosta, Peter Ezeah, Sara Gallardo, Veronica Montes de Oca, Hong Wang, Maëlenn Guerchet, Zhaorui Liu, Maria Sanchez, Peter Lloyd-Sherlock, Martin J Prince

**Affiliations:** Health Service and Population Research Department, King’s College London (Institute of Psychiatry, Psychology and Neuroscience), London, UK; Instituto de la Memoria y Desordenes Relacionados, Lima, Peru; Institute of Mental Health, Peking University, Beijing, China; National Institute of Neurology and Neurosurgery of Mexico, Autonomous National University of Mexico, Delegacion Tlalpan, Mexico City, Mexico; Nnamdi Azikiwe University Teaching Hospital, Nnewi, Anambra State Nigeria; Department of Sociology/Anthropology, Nnamdi Azikiwe University, Awka, Nigeria; School of International Development, University of East Anglia, Norwich, NR4 7TJ UK

**Keywords:** Long-term care, Ageing, Developing countries

## Abstract

**Background:**

In low or middle income countries chronic diseases are rapidly becoming the main cause of disease burden. However, the main focus of health policymakers has been on preventing death from cancer and heart disease, with very little attention to the growing problem of long-term needs for care (dependence). Numbers of dependent older people are set to quadruple by 2050. The economic impact of providing long-term care is likely to be substantial.

**Methods/design:**

The study uses mixed methods and draws on and extends the population-based surveys conducted by the 10/66 Dementia Research Group. We focus on two countries in Latin America (Peru and Mexico), China and Nigeria. The surveys comprised baseline surveys of health, socioeconomic circumstances and care arrangements, repeated three to four years later. We are going back to these households to make a detailed assessment of the overall economic status and the use of health services by all family members. We will compare households where: a) an older resident became dependent between baseline and follow-up (incident care), b) one or more older people were dependent at both time points (chronic care), b) c) no older residents had needs for care (control households) for household income, consumption, healthcare expenditure and economic strain. In each of the four countries we are carrying out six detailed household ‘case studies’ to explore in more depth the economic impacts of dependence, and the social relations between household members and others in their network.

**Discussion:**

The INDEP study will provide a detailed examination of the economic and social effects of care dependence in low and middle income settings. As the proportion of older people with needs for care rises rapidly in these countries, this neglected policy area is likely to become increasingly salient for families, communities and policymakers alike. Our detailed multilevel plans for dissemination will ensure that the study helps to put this important issue on the agenda for the international and national media, the public and researchers.

**Electronic supplementary material:**

The online version of this article (doi:10.1186/2193-1801-3-379) contains supplementary material, which is available to authorized users.

## Background

Between 2010 and 2050 the number of people aged 60 and over is projected to increase by one and a quarter billion, reaching 22 per cent of the world’s population. Of these, 81% will be living in Asia, Africa, Latin America or the Caribbean (DESA 2009). Along with population ageing, the prevalence of chronic diseases is rising quickly across low and middle income countries (LMIC), accounting, by 2010, for the majority of disease burden (Abegunde et al. 2007). Dependence is defined as ‘the need for frequent human help or care beyond that habitually required by a healthy adult’ (Harwood et al. 2004). The number of care-dependent older people in LMIC is forecast to quadruple by 2050 (Harwood et al. 2004). These demographic and epidemiological trends have profound implications for poverty reduction, gender relations and equity.

The 10/66 Dementia Research Group’s (10/66 DRG - http://www.alz.co.uk/1066/) program of population-based surveys on dementia, chronic diseases and ageing in Latin America, India, China and Nigeria has provided some of the first detailed data on the prevalence of care-dependence and the nature of care arrangements for older people in those regions. The prevalence of dependence ranged between 3% to 16% by site. Even after standardizing for the main chronic disease correlates of dependence, prevalence was higher in urban Latin America and China, compared with rural and less developed sites (Sousa et al. 2010). Differences in survival, and reporting biases may have accounted for this pattern. Dependence was more common in the older old, in women compared with men, and in those with least education. Among the chronic diseases dementia made the largest independent contribution to disability and dependence, followed by limb impairment, stroke and depression (Sousa et al. 2009, 2010). However, dependence is mainly characterised by comorbidity between cognitive, physical and mental disorders (Acosta et al. 2008; Llibre Rodriguez et al. 2008a; Uwakwe et al. 2009).

### Objectives

The 10/66 INDEP study described in this protocol will draw upon and extend the established set of international population-based surveys conducted by the 10/66 DRG (Prince et al. 2007). Between 2003 and 2008, the group conducted population-based surveys of 21,000 people aged 65 and over in 13 catchment areas in 9 LMIC (Mexico, Peru, Venezuela, Puerto Rico, Dominican Republic, Cuba, China, India and Nigeria) using the same protocol, definitions and assessment tools (Prince et al. 2007). An incidence phase (3 to 4 year follow-up) has now been completed in most of these sites. The 10/66 surveys are unique among the few such surveys of ageing conducted in LMIC in their in comprehensive coverage of the health (Prince et al. 2007; Llibre Rodriguez et al. 2008b; Guerra et al. 2009; Acosta et al. 2010) and socioeconomic status of older people, and their needs for care (Acosta et al. 2008; Liu et al. 2009; Uwakwe et al. 2009). Previous 10/66 surveys have focussed on the main carer/care-recipient dyad. In the INDEP study we will use detailed interviews of selected households in Mexico, Peru, China and Nigeria to assess the extent to which onset of dependence in older residents serves as an economic shock to households as a whole. We will also explore the effects on social relations in the household and beyond, paying particular attention to gender dynamics and decision-making.

### Research questions

1. Incident dependence, impoverishment and vulnerability.
  - To what extent is the onset of dependence associated with household impoverishment and economic vulnerability?
  - What are the pathways between care dependence and changes to household economic status?
  - What factors influence household resilience in the face of increased dependence?
  - To what extent does this depend on the external policy environment, including the reach of social protection and health services?
2. Intra-household effects and wider social dynamics.
  - How is the care burden for dependent older people distributed across household members and wider kinship networks?
  - What factors influence the distribution of the care burden inside and outside the household?
  - How are decisions about the allocation of care made and justified?
  - What are the effects on the carers and how do they perceive these care duties?
  - Are some care arrangements more effective than others at mitigating the economic effects of incident dependence?

## Methods

### Design

An integrated mixed methods approach will be used. We will measure the economic effects of care dependence by nesting the study within the pre-existing baseline and incidence waves of the 10/66 surveys in Peru, Mexico, and China, while in Nigeria we will supplement the baseline survey with a new incidence wave before implementing the INDEP study protocol. We will then use an incident case–control design, sampling within the well-characterised 10/66 survey samples to identify four groups of interest (see below) for more detailed household interview. The qualitative component will comprise a series of detailed household case studies comprising multiple key informant interviews and participant observation. Household selection will be guided by prior hypotheses and emerging data. We will also collect contextual data on relevant national policies, welfare and healthcare financing, and background information about study catchment areas including local health facilities and other relevant resources. This work will be based upon desk-based research: access to web-based resources such as policy documents, newspaper archives and national and regional government records. These data will be particularly pertinent in determining the impact of the external policy environment, including the reach of social protection and health services.

### Settings

The study will be based in 10/66 survey catchment areas in four countries; China, Peru, Mexico and Nigeria. At the baseline of the 10/66 survey, the Peru sites comprised urban catchment areas (1381 older people sampled in Lima Cercado and San Miguel in the capital city, Lima) and rural sites (n = 552 in Cerro Azul, Imperial, Nuevo Imperial, Quilmana, San Luis, San Vicente in Canete coastal province). In Mexico we also sampled urban (n = 1003 in six districts in Tlalpan, Mexico City) and rural (n = 1000 in nine villages in Morelos, a mountainous district 70 km from Mexico City) catchment areas. The urban site in China was Xicheng, close to Tiananmen Square (n = 1160), while the rural site comprised 14 villages in Daxing, a rural district 40 kilometres away (n = 1002). In Nigeria we sampled 1132 older people in seven mainly rural communities in Dunukofia, Anambra State.

### Selected households and participants

We defined care dependence as the needs for care that arise from difficulties in performing important tasks and activities related to daily living. These difficulties commonly occur among older people due to the interacting effects of multiple health problems: chronic physical conditions that affect different organ systems as well as co-morbid mental and cognitive disorders. Care needs of older people were ascertained at three time-points (baseline and incidence surveys and during the INDEP study interview). Data from baseline and incidence survey was used to categorise households in to the following three groups. Interviewers asked the person selected as a key informant even open-ended questions (what kind of help does X need inside of the home?; what kind of help does X need inside of the home?; who, in the family, is available to care for x?; what help do you provide?; do you help to organise care and support for x?; is there anyone else in the family who is also involved in helping?; what help do they provide?; what about friends and neighbours?; what help do they provide?) followed by an interviewer coding that the older person does not need care; needs care occasionally; or needs care much of the time. This judgment is further guided by an assessment of critical intervals of care; what do you think would be the longest period of time that X could manage by themselves, without help from others, supposing that they were living on their own? Those households where the older person(s) were categorised as “needs care much of the time” were those defined as incident/chronic care households (see definitions below). Key informants were selected by interviewers on the basis of who knew the older person best and who would be able to give the clearest and most detailed account of current circumstances and were usually co-resident or other family members.

1. Incident care households (where all older residents were independent at baseline, but in which one or more have become care dependent by the incidence survey).
2. Chronic care households (households containing one or more care dependent older people at baseline, who remained care dependent in the incidence survey).
3. Control households (where all older residents were independent at baseline, and remained so at the incidence survey).

All households meeting criteria for incident or chronic care were selected for inclusion in the INDEP study. In each site, control households equivalent in number to the sum of incident and chronic care households were selected in each site, at random from all those eligible, and batch matched to care households for the age of the oldest resident.

The designation of some care and control households will change, based upon changed circumstances since the last 10/66 (follow-up) survey. Where all index older people needing care have died (incident or chronic care households) the household will be re-designated as a ‘care exit’ household. Where all index older people have died in a control household, the household will be excluded from the study. Where index older people have moved to another physical location they will be followed up to the new household, and the change of location and household composition will be recorded.

### Quantitative research methodology

#### Data collection

For each selected household, we aim to conduct a household interview with a suitably qualified key informant (usually the self-defined head of household), brief interviews with each of the surviving index older people, and an informant interview for each older person to provide an independent perspective on their health and needs for care. The detailed household interviews are to be conducted masked to the household group status. Masking will not be possible in Nigeria, in which setting we will conduct incidence phase interviews selecting all incident and chronic care households, and every fourth control household for the INDEP study.

#### Measures

Household interviews for household income, consumption and assets have not been used in previous waves of the 10/66 survey. The questions for the INDEP study were developed from questionnaires used successfully in community research into social pensions, poverty and wellbeing in South Africa and Brazil (Lloyd-Sherlock et al. 2012). We further checked in a preparatory meeting with local investigators the relevance and comprehensiveness of questions regarding sources of income and types of expenditure, and adjusted the phrasing of questions for each country to reflect the local systems.

Interviews were piloted in local settings. The primary aim of piloting was to assess the acceptability of the length of interviews. Length of household interviews was variable (depending on number of household members) but was generally found to be acceptable. Minor changes to syntax were made in response to piloting and in some cases additional clarifications were added to ensure that the meaning of questions reflected that agreed upon in the preparatory meeting.

The detailed household interview comprises:

1. Economic evaluation
  - A household assets index covering household goods and amenities (telephone or mobile phone, stove, electricity supply, television, radio or stereo, refrigerator, sewing machine, bicycle, computer, and motor vehicles), and ownership of land, property and livestock.
  - Assets in savings or investments (bank or savings account, stocks or shares)
  - Total monthly equivalent net household income, calculated by ascertaining the amounts and sources of all regular incomes (20 items), and the identity of recipients. Total income will be divided by the modified OECD equivalence scale (1.0 for the first adult, 0.5 for all other adults, and 0.3 for children) to account for economies of scale.
  - Consumption, 25 items eliciting food consumption (the value or cost of all food consumed at home and outside of the home), household expenses and other personal expenditure (Angelini et al. 2008), also divided by the OECD equivalence scale. For each expenditure item we enquire whether this is about the same, more, less or much less than in a typical month one year previously.
  - Out of pocket expenditure on all health and home care services in the last three months, for each household member.
  - Household debt and loans, and other indicators of financial strain. These included; asking for help from friends or relatives, an employer, a religious organisation, or charity; borrowing from a bank, moneylender or loan shark; cutting down on food consumption; trying to find extra work; running up an account with a shop; applying for a grant; apply for food parcels or vouchers; drawing on savings, selling stocks or shares; any other action to address the financial difficulty.
  - Subjective assessment of overall financial status; How would you rate the financial situation of this household at present? Is it very good, good, average, bad or very bad? How would you rate the financial situation of the household compared to three years ago? Is it better, the same or worse than three years ago?
2. Household composition and roles
  - Current household composition, and all changes since baseline interview (with reference to household composition recorded at that time).
  - Current economic activity of all household members (full-time education, full or part-time employment/nature of occupation, seeking work, disabled, retired, homemaker), reasons for not being in paid work (including providing care to children or older household residents) and changes in status since baseline interview.
  - Health status of all household residents, needs for care arising from long-term illness or disability, and the identity of the main caregiver for all residents needing care.

The main purpose of the brief interview with each index older person is to update information on their health status since the last 10/66 survey, through self-reported health and disability (World Health Organisation Disability Assessment Scale (WHODAS 2.0) (WHO 2010). We also collect information on personal income, intergenerational reciprocity (gifts or transfers of money to other household members, and care or supervision of children or others), decision-making autonomy, needs (comfort and shelter, food, medical care, clothes and other necessities of daily life) met and unmet, and life satisfaction. If the index older person lacks capacity to provide this information we conduct the interview with a suitably qualified proxy informant.

The main purpose of the interview with a suitably qualified key informant for each older person is to assess their current needs for care. The interview is based upon the methods used in the 10/66 surveys, as outlined previously in the description of the selection of households for the INDEP study. In the INDEP study, we will look at the content of the care needs in more detail. For those older people requiring care, we enquire about the daily time spent assisting with communication, transport, dressing, eating, grooming, toileting, bathing, and general supervision. We also establish the identities of all household residents providing care for the older person, and whether they had stopped education or work to provide care.

#### Analyses

We will use multi-level mixed effects analyses (residents nested within households) to test the hypotheses that, controlling for baseline household composition and assets:

1. Incident and chronic care households have lower annual equivalised net household incomes and lower total food consumption than control or care exit households
2. Children (aged 15 and under) who were resident at baseline in chronic and incident dependence households are less likely to have completed secondary education (12 years) and will have completed fewer total years of education than children in control households
3. Out-of-pocket healthcare and homecare costs will be higher in incident and chronic care households than control or care exit households
4. That effects 1 to 3 above are mediated by levels of disability and total person hours of care and supervision required by older residents
5. That effects 1–3 above will be modified by household size (larger households being better placed to absorb shocks), the age of the main carer (smaller effects when the carer is aged 65 or over), and by indicators of social protection (pensions, cash transfers from outside of the household, health insurance)

Quantitative analysis will also be used to explore factors associated with particular patterns of household care allocation. Inter alia, these will include household factors (e.g. household composition, socio-economic status), those related to the dependent older person (e.g. sex, pension status and other income, relationship to household head) and those relating to the main carer (e.g. employment status, age, relationship to care-recipient). Particular attention will be given to factors associated with use of paid care by non-family members.

Power analysis suggests that we will have 90% power (at 95% confidence) to detect small to medium effect sizes (0.47 to 0.65) on e.g. consumption, income and healthcare expenditure when comparing dependent and control households where, as for most sites, the numbers of household in each group ranges between 100 and 250. For Nigeria, where numbers of households in each group are likely to be smaller, between 55 and 70 households in each group would permit detection of moderate to large effect sizes, (0.78 to 0.89) at 90% power or (0.66 to 0.77) at 80% power.

We will analyse data from completed questionnaires only. Experience from other 10/66 studies suggests that the level of missing data within otherwise complete questionnaires is likely to be low. We will analyses reasons for non-completion of interviews using data from baseline and incidence questionnaires.

### Qualitative research methodology

Case study households (approx six per site) will be purposively sampled from the quantitative survey. Control households will not be included in the qualitative analysis. For each household, interviews will be conducted separately with several key informants including dependent older people (where feasible), the main carer, any other household or non-household members identified as playing a significant role in caring for the dependent older person, the household head and other key decision makers. This will yield a set of detailed and comprehensive household case studies nested within the larger quantitative study.

Guidance for qualitative interviews was developed iteratively. Following early pilot interviews carried out in Peru, it was decided that interviews will be done in a narrative style, allowing interviewees to “tell a story” about the older person’s care needs, the impact of this upon the household and how the household has coped with these changes. Experiences from pilot interviews in Peru suggested that this interviewing style would elicit the richest data due to the close resemblance of the interviews to how participants might discuss their experiences about the onset of dependency outside of the context of the study. Interviewers will be asked to make notes about key events, so that they are then able to ask about decision-making and changes to household finances related to these events. Interviewers will also complete a family tree, mapping the key relationships within and outside of the household.

The development of the qualitative methodologies has been iterative and informed by initial qualitative and quantitative data as well as interviewer’s early experiences of pilot interviews. The qualitative team met in London in May 2013 to discuss emerging themes from pilot data (by this time, interviews with at least one household in each country had been carried out) and to plan the main phase of data collection. The following key household characteristics were identified as being of particular interest in relation to the initial research questions.

- Chronic poverty i.e. households with few economic resources wherein this situation has been long-term rather than short-term
- Incident poverty i.e. short-term reduction in economic wealth, often due to illness, jobloss, household changes
- Large households i.e. those with extended families living in the same household
- Small households i.e. where older people live alone or with a spouse only
- Households with substantial difficulties i.e. substance abuse, debt, violence
- Households that seem to be coping well with the challenges of dependency and/or economic poverty
- Female-headed households
- Households where there is more than one dependent person i.e. older person + others who need substantial care- young children, others with disability
- Other households that stand out/were memorable for some reason

Criteria were selected after consideration of the INDEP themes and research questions, discussions of emergent findings from pilot interviews and meeting with members of staff from HelpAge International and ADI (carried out 13^th^-17^th^ June, 2013, London).

It was also decided to optimise the mixed methods structure of the INDEP study by gathering further background data from the quantitative teams by carrying out focus group discussions. Quantitative teams were asked to discuss households that particularly stood out in relation to the key characteristics identified from pilot data. This data will be used in combination with quantitative datasets to identify households for interview in the main phase of qualitative data collection.

Although based upon iteratively developed topic guides, interviews will be receptive to other considerations that informants consider to be significant. Data will be fully transcribed and translated, and then managed in NVivo7. Analysis will be informed by the initial research questions, but will be strongly inductive since a key aim is to explore informants’ own perceptions of and explanations for responses to incident dependence. The anonymised data sets will be archived in the Economic and Social Data Service.

### Project resources and training

Field work in each country is led by a research coordinator, with quantitative interviews (household and older person interviews) conducted by a team of four to six appropriately trained local research assistants. After initial tracing of the index older persons (defining a household as control, incident or chronic care) the research assistant (blinded to household status) contacts the research coordinator for unblinded advice as to how to proceed.

### Data management

Questionnaire data from the household and older person interviews is checked for completeness, and then double data entered on to specially developed EPIDATA data entry systems, including range and consistency checks. All qualitative data from open-ended interviews is recorded on digital audio recorders, transcribed in the local language (generally Spanish, Mandarin or Igbo), and then translated into English.

### Ethical Issues

The INDEP study protocol has been approved by King’s College London Research Ethics Committee and relevant local authorities in each study site. It is to be expected that a significant proportion of the older people who are potential participants in the study will currently have dementia. We will use an approach similar to that used previously in 10/66 studies: if the older person lacks capacity to consent, the next of kin will be asked to provide signed assent. Participation will be subject to the older person not showing signs of distress or dissent when the information sheet is read to them. For each household, the index older person or persons are first approached for consent for an individual and informant interview, and to nominate a suitable key informant for the household interview. If they do not consent, the household is excluded.

## Results

### Background descriptive data on INDEP households, participants and countries

#### Caregiving context for dependent older people (10/66 baseline surveys)

The prevalence of dependence in the baseline survey, and the caregiving context for dependent older people in the four countries (seven sites) selected for the current study is summarised in Table 1. The norm is to live with adult children or children-in-law. Three generation households, including children under the age of 16 are common in all sites other than urban China. Caregiving is mainly done by women, although men were more likely to be nominated as the principal carer in China. Carers often report giving up or cutting back on work to care, although this arrangement was less common in Lima and Beijing, where paid carers are often employed. Caring roles are commonly shared with other informal carers, other than in China, where an isolated main carer seems to be the norm. Caregiving, as in high income countries, is often associated with considerable psychological strain (Patel and Prince 2001; Shaji et al. 2003; Prince and Dementia Research 2004).

**Table 1:** **Care-giving context for dependent older people selected for participation in INDEP**

	Peru	Peru	Mexico	Mexico	China	China	*Nigeria
	Urban	Rural	Urban	Rural	Urban	Rural	
Prevalence of dependence	135/1381 (9.7%)	26/550 (4.7%)	114/1003 (11.4%)	82/1000 (8.2%)	183/1160 (15.8%)	54/1002 (5.4%)	233/872 ()
Caregiving context for dependent older people	N = 135	N = 26	N = 114	N = 82	N = 183	N = 54	N = 228
Household composition							
Living alone	0.0%	7.7%	8.8%	4.9%	2.7%	0.0%	
Living with spouse only	5.2%	3.8%	14.9%	9.8%	26.8%	11.1%	
Co-resident adult children	69.6%	76.9%	69.3%	78.0%	50.3%	87.0%	
Co-resident children under 16	29.6%	38.5%	34.2%	51.2%	13.7%	63.0%	
Mean household size (SD)	4.7 (2.1)	5.3 (3.0)	4.1 (2.4)	4.3 (2.3)	3.0 (1.4)	4.9 (1.6)	
Principal carer characteristics							
Spouse	18.5%	26.9%	16.7%	15.9%	38.8%	38.9%	13.7%
Child or child-in-law	40.0%	50.0%	73.7%	65.8%	43.2%	59.3%	68.0%
Non-relative	25.2%	3.8%	3.6%	0.0%	16.4%	1.9%	1.4%
Female carer	85.9%	88.5%	83.3%	81.7%	67.2%	50.0%	63.2%
Care arrangements							
Carer has cut back on work to care	16.3%	23.1%	25.4%	36.6%	3.8%	48.1%	39.2%
Additional informal carer or carers	45.9%	57.7%	55.3%	58.5%	7.1%	22.2%	66.5%
Paid carer	33.3%	7.7%	3.5%	1.2%	45.4%	1.9%	2.1%

*Incidence data collection is still underway in Nigeria and hence only selected variables presented here.

#### INDEP households

Across the three countries (Peru, Mexico and China) that have completed baseline and follow-up 10/66 surveys, we were able to select 491 incident care households, 190 chronic care households, and 669 age-matched control households (see Table 2 for details). Of these households, 374 were in Peru, 468 in Mexico and 512 in China. In the 1350 households, 1781 residents aged 65 years and over had completed baseline and follow-up survey interviews. There were insufficient control households with residents in the oldest age groups to age match directly in China; otherwise there were equal numbers of control households in each age group (judged by the age of oldest resident) as the sum of incident and chronic care households. In each site, household wealth (assets) and composition was quite similar between incident and chronic care and control households (Table 3). However, there was a general trend towards a high proportion of single older person households, and a smaller household size among the control households, which differences were statistically significant for urban China. Three generation households, including children under the age of 16, were more common in Latin American than Chinese sites, and particularly uncommon in urban China.

**Table 2:** **Households included in INDEP**

	Incident care households (older residents)	Chronic care households (older residents)	Control households (older residents)	TOTAL households (older residents)
Peru (urban)	87 (125)	51 (75)	138 (178)	276 (378)
Peru (rural)	38 (46)	11 (12)	49 (55)	98 (113)
Mexico (urban)	84 (109)	37 (53)	121 (148)	242 (310)
Mexico (rural)	85 (106)	26 (40)	111 (133)	222 (279)
China (urban)	124 (175)	56 (75)	168* (233)	348 (483)
China (rural)	73 (99)	9 (11)	82 (108)	164 (209)
TOTAL	491 (660)	190 (266)	669 (855)	1350 (1781)

*there were insufficient control households with older participants to age match directly in urban China.

**Incidence data collection is still underway in Nigeria and hence not presented here.

**Table 3:** **Household assets and composition (at follow-up interview) by household selection characteristics**

*Assets, median (25 ^th^, 75 ^th^ centile)	Incident care	Chronic care	Control	Kruskal-Wallis test, p-value
China urban	6 (5–6)	6 (5–6)	6 (5–6)	0.24
China rural	6 (5–6.5)	6 (4.5-7)	6 (5–6)	0.71
Peru urban	6 (6–6)	6 (6–6)	6 (6–6)	0.63
Peru rural	5 (4–6)	5 (5–6)	5 (5–6)	0.51
Mexico urban	6 (6–7)	6 (6–7)	6 (6–7)	0.66
Mexico rural	4 (3–5)	5 (3–6)	4 (3–6)	0.27
**Household composition**				
**Older person living alone, n (%)**	**Incident care**	**Chronic care**	**Control**	**Chi-sq, p-value**
China urban	1 (0.8%)	2 (3.6%)	22 (13.1%)	23.5, <0.001
China rural	4 (5.5%)	0 (0.0%)	9 (11.0%)	2.7, 0.62
Peru urban	3 (3.4%)	2 (3.9%)	7 (5.1%)	3.7, 0.45
Peru rural	4 (10.5%)	1 (9.1%)	5 (10.2%)	3.1, 0.54
Mexico urban	8 (9.5%)	5 (13.5%)	25 (20.7%)	6.3, 0.18
Mexico rural	11 (12.9%)	2 (7.7%)	17 (15.3%)	4.4, 0.36
**Co-resident children <16, n (%)**	**Incident care**	**Chronic care**	**Control**	**Chi-sq, p-value**
China urban	9 (7.3%)	2 (3.6%)	10 (6.0%)	0.9, 0.63
China rural	14 (19.2%)	1 (11.1%)	21 (25.6%)	1.6, 0.45
Peru urban	34 (39.1%)	17 (33.3%)	53 (38.4%)	0.5, 0.77
Peru rural	19 (51.4%)	4 (36.4%)	16 (32.7%)	3.1, 0.21
Mexico urban	25 (29.8%)	16 (43.2%)	30 (24.8%)	4.7, 0.10
Mexico rural	28 (32.9%)	6 (23.1%)	25 (22.5%)	2.9, 0.24
**Household size, median (25** ^**th**^ **, 75** ^**th**^ **centile)**	**Incident care**	**Chronic care**	**Control**	**Kruskal-Wallis test, p-value**
China urban	3 (2–4)	3 (2–3)	2 (2–3)	0.03
China rural	4 (2.5-5)	3 (2–4.5)	4 (2–5)	0.69
Peru urban	4 (3–6)	5 (3–6)	4 (2–6)	0.11
Peru rural	4 (2–5)	4 (3–5)	3 (2–5)	0.27
Mexico urban	4 (2–5)	3 (2–6)	3 (2–5)	0.14
Mexico rural	3 (2–5)	3.5 (2–5)	3 (2–4)	0.11

*Number of assets in the household out of a possible total list of seven (TV, fridge/freezer, mains water, electricity, telephone, plumbed toilet, plumbed bathroom).

**Incidence data collection is still underway in Nigeria and hence not presented here.

**Figure 1:**
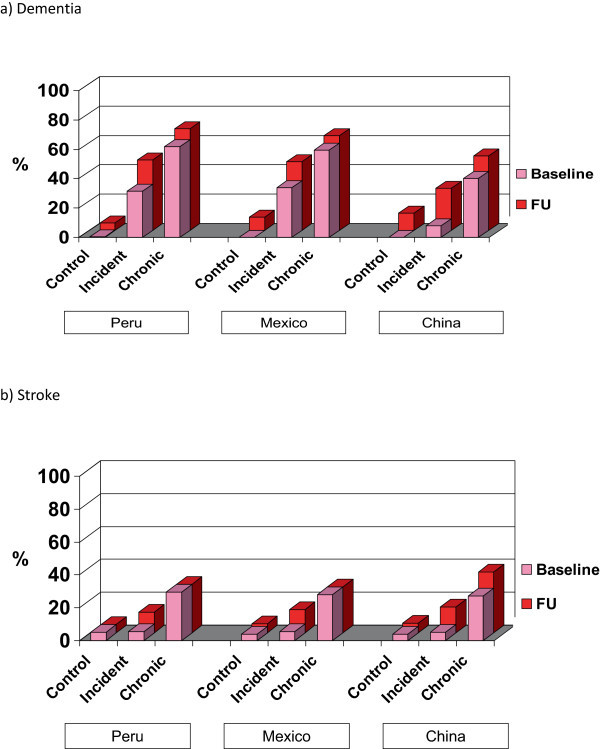
**Prevalence of dementia (a) and stroke (b) among index older people by household selection status and country at baseline and follow-up survey 1a) Dementia.**

Despite age matching, those needing care in the incident and chronic dependence households were around two years older on average than participants in the control households, none of whom had had needs for care (Table 4). This is explained by the fact that matching was carried out on the age of the oldest household member, in five year bands, and participants not needing care in the ‘care’ households were excluded from this individual level analysis. Household groups were reasonably well matched in terms of gender and level of education. Otherwise, the characteristics of the index older people mainly reflected and validated the selection criteria. In the incident households, those needing care at follow up had low disability (WHODAS 2.0) mean scores at baseline, rising to high levels (similar to those seen in the chronic households at baseline) by follow-up. In the chronic dependence households, mean disability scores were high throughout, even higher at follow-up than at baseline. In the control households mean disability scores were close to zero throughout. The proportion of index older people requiring ‘much’ care increased slightly from baseline to follow-up in the chronic care households, while the proportion in incident care households at follow-up was slightly lower than that at baseline in the chronic care households. Dementia was the most common disabling chronic condition among index older people in incident and chronic care households, and the condition that most clearly distinguished care and control households. The prevalence rose from baseline to follow-up survey, by which time up to one half of index older people in the incident care households, and two-thirds in the chronic care households were affected (see Figure 1a). By contrast there was only one dementia case among residents of control households at baseline, while between 5 and 12% were affected at follow-up. A similar pattern was seen for stroke, but with a lower prevalence and a less marked distinction between care and control households (see Figure 1b). Patterns were consistent across urban and rural catchments in all sites, therefore the data presented in Table 4 is described by country.

**Table 4:** **Characteristics of index older people resident in incident dependence, chronic dependence and control households**

	Incident care	Chronic care	Control	
PERU	126	68	233	
Age	80.6 (8.2)	80.4 (7.9)	77.8 (6.6)	7.3, 0.001
Gender (male)	40 (31.7%)	22 (32.4%)	96 (41.2%)	3.9, 0.14
Educational level (did not complete primary)	38 (30.6%)	14 (20.9%)	49 (21.2%)	4.3, 0.11
Mean change in WHODAS disability score from baseline	+21.8 (31.0)	+10.0 (30.4)	+1.7 (14.8)	29.9, <0.001
Needs for care at baseline (much care)	No needs for care	35 (51.5%)	No needs for care	-
Needs for care at FU (much care)	53 (42.1%)	48 (70.6%)	No needs for care	14.4, <0.001
MEXICO	175	64	281	
Age	77.8 (6.8)	78.8 (6.7)	76.8 (6.0)	3.2, 0.04
Gender	65 (37.1%)	14 (21.9%)	106 (37.7%)	6.0, 0.05
Educational level (did not complete primary)	45 (25.7%)	11 (17.2%)	77 (27.4%)	2.9, 0.24
Mean change in WHODAS disability score from baseline	+28.2 (32.0)	+11.5 (35.5)	+4.2 (19.0)	44.7, <0.001
Needs for care at baseline (much care)	No needs for care	36 (56.3%)	No needs for care	-
Needs for care at FU (much care)	58 (33.1%)	35 (54.7%)	No needs for care	9.2, 0.02
CHINA	212	70	341	
Age	75.3 (6.1)	75.9 (6.2)	73.7 (5.3)	7.3, 0.001
Gender	76 (35.8%)	24 (34.3%)	141 (41.3%)	2.3, 0.32
Educational level (did not complete primary)	84 (39.6%)	36 (51.4%)	203 (59.5%)	20.8, <0.001
Mean change in WHODAS disability score from baseline	+33.7 (29.9)	+16.1 (30.7)	+4.2 (10.1)	123.0, <0.001
Needs for care at baseline (much care)	No needs for care	45 (64.3%)	No needs for care	-
Needs for care at FU (much care)	106 (50.0%)	53 (75.7%)	No needs for care	14.1, <0.001

*Incidence data collection is still underway in Nigeria and hence not presented here.

#### ***Pensions, healthcare insurance and financing in the INDEP countries (see online resource – Additional file***1***: Table S1)***

All four of the upper and lower middle income countries included in the INDEP study have undergone substantial pension reform since the 1990s, aiming to increase coverage through contributory, social and hybrid schemes as well as seeking to ensure fiscal sustainability in the coming decades (Lavigne 2013; Dostal 2010; Carranza et al. 2012; Vilela 2013). Despite these efforts, the proportion of older people in receipt of any pension remains low: 60 percent or less (International; Scott 2008; Dostal 2010; Rofman and Oliveri 2012). Social pensions (or hybrid social/contributory in the China) are available in all four countries but in the case of Mexico and Nigeria, these are regional schemes sponsored by state governments and therefore dependent upon residency within a particular town or region. Levels of social pension range from 4.8 percent of average national income in rural Mexico (International) to 50 percent of average national income in Nigeria (International). In Nigeria, although the two social pension schemes offered are generous, only small numbers of older people are currently in receipt of these benefits- because of difficulties with roll-out in Ekiti State and because the pension available in Osun state is targeted only at the most vulnerable older people (International). In China, parallel schemes consisting of national and local government as well as individual contributions are available to those registered in urban or rural zones (Vilela 2013). High levels of employment within the informal labour sector (Fasanya and Onakoya 2012; OECD 2013) and contributory pensions that have been historically limited to particular sectors of the formal market have contributed further to low overall pension coverage.

In all four countries, the majority of health expenditure is borne by service-users and their families. Extensive policy reform in Mexico means that by end of 2011, 98 percent of Mexicans were enrolled in health insurance schemes covering a basic package of care (but not treatment and care for many chronic conditions) (International; Knaul and Frenk 2005; Knaul, et al. 2012). In Peru, the central government provides basic services to those otherwise uninsured, whilst EsSalud (funded by employer contributions) caters for those employed in the formal sector (Alcalde-Rabanal et al. 2011). Problems with supply (resulting in long waiting lists) mean that private sector healthcare utilisation remains high (Alcalde-Rabanal et al. 2011). In China, parallel systems, funded by a combination of central and local government and individual contributions support variable reimbursements for costs of health service use for urban and rural residents (Li et al. 2011; Lloyd-Sherlock et al. 2012). In Nigeria, despite policy reforms in 2005, aimed at increasing health insurance coverage, less than 3 percent of the population are insured (mostly government employees) and more than 95 percent of the costs of healthcare costs are paid be service-users and their families(Odeyemi and Nixon 2013).

## Discussion

### Relevance of INDEP

Little is known about the lives of older dependent people in LMICs and the effects of the older person’s health and social care needs upon the households in which they live. Evidence from our recent cross-sectional survey in Nigeria suggested that although the only recourse for older dependent people was to live with or to be directly provided for by their family, families were not always in a position be able to ensure adequate social protection (Acosta et al. 2010). Findings from this and other 10/66 sites suggested that caregiving may be associated with adverse economic impacts upon the household, including increased healthcare expenditure and the caregiver (usually female) cutting back on paid work in order to care (Acosta et al. 2008, 2010; Liu et al. 2009; Uwakwe et al. 2009). The increased global health focus on chronic disease is mainly concerned with preventing cardiovascular and cancer mortality (Yach et al. 2004; Beaglehole et al. 2008), rather than addressing the long-term consequences of other disorders more associated with disability and dependence(Prince et al. 2008; Sousa et al. 2009, 2010). In most LMICs, long-term care policy continues to be premised on the questionable assumption that it can be left to informal provision (Brodsky et al. 2003; Prince et al. 2008).

The INDEP study will provide a detailed picture of social and economic consequences of dependence in older age in four low and middle income countries. The incident case–control design of the INDEP study will enable us to examine the economic impact of chronic and more recent onset of needs for care among older people, whilst controlling for age. We will be able to investigate the mediating effects of disability and the modifying effects of household size, age of main carer and social protection upon the relationship between care dependence and the economic health of the household. The qualitative elements of INDEP will enable us to examine the mechanisms for any observed associations between care dependence and household impoverishment, including factors that support economic resilience.

The pace of demographic, social and economic change in low, and particularly middle income countries will have a transformative effect on informal long-term care arrangements. Policy development in this area, bolstering family care and providing supplementary or alternative support, needs to be informed by a better understanding of current systems. There is currently little information on the effects of caregiving on the status of female carers within the household. Research from Mexico (Varley and Blasco 2003) shows that in three generation households a matriarch’s power over her daughter-in-law can weaken as she becomes care dependent and the daughter in law becomes empowered as the *de facto* matriarch. Alternatively, withdrawing from paid labour may weaken women’s status within the household (Sen 1987; Agarwal 1994). However, studies of intra- household gender relations do not always show direct links between independent income and a woman’s status (Farah-Quijano 2010). We will examine their current situation and how caregiving affects their wider lifetime experiences of employment and unpaid caring. We will examine how carers combine these responsibilities with other duties, including care for other family members, such as children. Does the double burden of paid work and housework become a treble burden? (there are thinly evidenced claims for this in Malaysia (Norzareen and Nobaya 2010)). We will also purposively select some households for detailed case study that have employed a paid non-family carer, probably from Lima and Beijing where this practice is common. Drawing on the literature concerned with domestic service more generally (Parrenas 2001; Lund and Budlender 2009; YANG 2009), the interviews will assess whether elder care can be considered as an extension of paid domestic tasks such as cleaning and childcare or is qualitatively different. For example, whether paid carers have formal qualifications, whether they recruited in similar ways to other domestic workers, and the potential for exploitation of carers.

National and local level situational data will ensure that study findings are locally relevant, hence for example discussion of factors that promote financial strain or economic resilience will be considered in the context of local and national policies and provision of services and social protection. This will enable us to consider the extent to which the healthcare and social protection environment mediates the economic and social effects of dependence at the household level.

### Methodological issues

The INDEP study sample is derived from participants in the previous waves of the 10/66 Dementia Research Group surveys. Response rates for baseline surveys were generally high (74 to 98 percent). At the incidence phases, the non-response rates (not traced or refused) were mostly modest (less than 18 percent in all but urban Peru where non-response was 29 percent) and non-differential with respect to most participant characteristics at baseline. Catchment area sampling has enabled us to develop strong links with local communities as well as with individual households. Nevertheless, there is likely to be significant further attrition, from household mobility, and refusal for a third wave of interviewing.

The detailed household interviews for household income, consumption and assets have not been used in previous waves of the 10/66 survey. The questions were based upon interviews used successfully in community research in South Africa and Brazil (Lloyd-Sherlock et al. 2012). We further checked in a preparatory meeting with local investigators the relevance and comprehensiveness of questions regarding sources of income and types of expenditure, and adjusted the wording of questions for each country to reflect the local systems. Nevertheless, there remains some uncertainty as to whether households are willing to share this information, whether a single key informant will be able to provide accurate information regarding all residents, and whether responses may systematically under-report or over-report the correct level of these economic indicators. It will therefore be important to examine carefully the internal consistency of the data generated, and to test concurrent validity (associations between household income, consumption and assets, and associations with socioeconomic indicators and determinants gathered in previous 10/66 surveys).

Households were selected for inclusion and household group status determined upon the basis of the needs for care of the older adults who lived there, at the time of the 10/66 baseline and follow-up surveys. While the descriptive data presented in this paper is reassuring, with respect to levels of disability and prevalence of dementia and stroke at baseline and follow-up among older residents in the three groups of households, misclassification remains a possibility. Needs for care are sometimes difficult to ascertain in less developed settings, owing to low awareness about underlying health conditions, respect for older people, and, often, routine provision of high levels of support regardless of ‘need’. Also, it is possible that in the interval between the follow-up survey and the INDEP study, older residents needing care in the care households may have recovered function, and, more likely, those in the control households may have developed needs for care. Although our *a priori* hypotheses are linked to the original household group classification, we will carry out sensitivity analyses accounting for changes in needs for care identified in the INDEP survey older person informant interview.

### Dissemination

We will use a multilevel approach so that our findings draw attention to dependence and its economic consequences among policymakers, the media and the public. It is essential that how to address the growing numbers of older people with long-term needs for care becomes part of the broader debates around population ageing. Our communications plan includes strategies for engagement with national and international policy-makers, the media and the public, as well as with the scientific community. Our partnerships with ADI (Alzheimer’s Disease International) and HelpAge International are crucial in our aim of maximising the impact of our study findings: communications about study progress and findings will be disseminated via these organisations’ networks. Research teams in each country will establish a communications working group to oversee national communications, working closely with key stakeholders to develop plans to engage with journalists and media agencies. Oral testimonies, film and photography will be used to ensure that the voices and experiences of those most affected- older people, their families and communities are powerfully communicated. Through policy briefs and working papers, we will disseminate key project findings to specific groups of policymakers. Study findings will be published in high impact and specialist peer-reviewed journals. We will use the 10/66 website as a repository for all study documents (oral testimonies, films, protocols, policy-briefs, scientific publications etc.), so that these are easily and rapidly accessible to all. Email addresses are registered for downloads, allowing us to add contacts for future disseminations. Project updates will also appear in a regular newsletter (an established part of the 10/66 project).

## Electronic supplementary material

Additional file 1: Table S1: Pensions, Healthcare Insurance and Healthcare Financing in Mexico, Peru, China and Nigeria (International; Carranza, et al 2012; Tretreault et al 2012; Scott, 2008; Knaul et al., 2012; Knaul & Frenk, 2005; International; Lavigne 2013; Rofman & Oliveri, 2012; International; Alcalde-Rabanal, et al., 2011; Vilela, 2013; Li, et al., 2011; Liu, 2012; Adebayo & Dada, 2012; Dostal, 2010; Odeyemi & Nixon, 2013). (DOCX 28 KB)

## References

[CR1] Adebayo AI, Dada R (2012). Pension Crisis in Nigeria: Causes and Solutions. IOSR Journal of Applied Chemistry (IOSR-JAC).

[CR2] Abegunde DO, Mathers CD, Adam T, Ortegon M, Strong K (2007). The burden and costs of chronic diseases in low-income and middle-income countries. The Lancet.

[CR3] Acosta D, Rottbeck R, Rodríguez G, Ferri CP, Prince MJ (2008). The epidemiology of dependency among urban-dwelling older people in the Dominican Republic; a cross-sectional survey. BMC Public Health.

[CR4] Acosta D, Rottbeck R, Rodríguez JG, González LM, Almánzar MR, Minaya SN, Ortiz Mdel C, Ferri CP, Prince MJ (2010). The prevalence and social patterning of chronic diseases among older people in a population undergoing health transition. A 10/66 Group cross-sectional population-based survey in the Dominican Republic. BMC Public Health.

[CR5] Agarwal B (1994). A Field of one’s own: Gender and Land Rights in South Asia.

[CR6] Alcalde-Rabanal JE, Lazo-González O, Nigenda G (2011). The health system of Peru. Salud Pública de México.

[CR7] Angelini V, Brugiavini A, Weber G, Börsch-Supan A (2008). Consumption. Health, Ageing and Retirement in Europe (2004-2007) - Starting the Longitudinal Dimension.

[CR8] Beaglehole R, Epping-Jordan J, Patel V, Chopra M, Ebrahim S, Kidd M, Haines A (2008). Improving the prevention and management of chronic disease in low-income and middle-income countries: a priority for primary health care. The Lancet.

[CR9] Brodsky J, Habib J, Hirschfeld M (2003). Long-term care in developing countries: ten case-studies.

[CR10] Carranza L, Melguizo Á, Tuesta D (2012). Matching Contributions in Colombia, Mexico, and Peru: Experiences and Prospects. Matching Contributions for Pensions.

[CR11] Desa U (2009). World Population Prospects: The 2008 Revision. Department for Economic and Social.

[CR12] Dostal JM (2010). Nigerian Pension Reform 2004–2010: Great Leap or Inappropriate Policy Design?.

[CR13] Farah-Quijano MA (2010). Bargaining Over Money and Land: Changing Intra-Household Gender Relations in Rural Colombia.

[CR14] Fasanya I, Onakoya ABO (2012). Informal sector and employment generation in Nigeria: an error correction model. Res Human Soc Sci.

[CR15] Guerra M, Ferri CP, Sosa AL, Salas A, Gaona C, Gonzales V, de la Torre GR, Prince M (2009). Late-life depression in Peru, Mexico and Venezuela: the 10/66 population-based study. Br J Psychiatry.

[CR16] Harwood RH, Sayer AA, Hirschfeld M (2004). Current and future worldwide prevalence of dependency, its relationship to total population, and dependency ratios. Bulletin of the World Health Organization.

[CR17] HelpAge International “Country Fact Sheet, China”Retrieved 18/10/13, 2013, from http://www.pension-watch.net/pensions/country-fact-file/china/

[CR18] HelpAge International “Country Fact Sheet, Mexico”Pension Watch Retrieved 18/10/2013, 2013, from http://www.pension-watch.net/country-fact-file/mexico/

[CR19] HelpAge International “Country Fact Sheet, Nigeria”Retrieved 18/10/2013, 2013, from http://www.pension-watch.net/pensions/country-fact-file/nigeria/

[CR20] HelpAge International “Country Fact Sheet, Peru”Retrieved 18/10/2013, 2013, from http://www.pension-watch.net/pensions/country-fact-file/peru/

[CR21] Knaul FM, Frenk J (2005). Health insurance in Mexico: achieving universal coverage through structural reform. Health Aff.

[CR22] Knaul FM, González-Pier E, Gómez-Dantés O, García-Junco D, Arreola-Ornelas H, Barraza-Lloréns M, Sandoval R, Caballero F, Hernández-Avila M, Juan M, Kershenobich D, Nigenda G, Ruelas E, Sepúlveda J, Tapia R, Soberón G, Chertorivski S, Frenk J (2012). The quest for universal health coverage: achieving social protection for all in Mexico. Lancet.

[CR23] Lavigne M (2013). Social protection systems in Latin America and the Caribbean: Peru.

[CR24] Li C, Yu X, Butler JR, Yiengprugsawan V, Yu M (2011). Moving towards universal health insurance in China: performance, issues and lessons from Thailand. Social Science & Medicine.

[CR25] Liu H, Zhao Z (2012). Impact of China's urban resident basic medical insurance on health care utilization and expenditure (No. 6768).

[CR26] Liu Z, Albanese E, Li S, Huang Y, Ferri CP, Yan F, Sousa R, Dang W, Prince M (2009). Chronic disease prevalence and care among the elderly in urban and rural Beijing, China - a 10/66 Dementia Research Group cross-sectional survey. BMC Public Health.

[CR27] Llibre Rodriguez J, Valhuerdi A, Sanchez II, Reyna C, Guerra MA, Copeland JRM, McKeigue P, Ferri CP, Prince MJ (2008a). The prevalence, correlates and impact of dementia in Cuba. Neuroepidemiology.

[CR28] Llibre Rodriguez JJ, Ferri CP, Acosta D, Guerra M, Huang Y, Jacob KS, Krishnamoorthy ES, Salas A, Sosa AL, Acosta I, Dewey ME, Gaona C, Jotheeswaran AT, Li S, Rodriguez D, Rodriguez G, Kumar PS, Valhuerdi A, Prince M, 10/66 Dementia Research Group (2008b). Prevalence of dementia in Latin America, India, and China: a population-based cross-sectional survey. Lancet.

[CR29] Lloyd-Sherlock P, Barrientos A, Moller V, Saboia J (2012). Pensions, poverty and wellbeing in later life: Comparative research from South Africa and Brazil. Journal of Aging Studies.

[CR30] Lund F, Budlender D (2009). Paid Care Providers in South Africa: Nurses, Domestic Workers and Home-Based Care Workers.

[CR31] Norzareen M, Nobaya A (2010). Women of the sandwich generation in Malaysia. Eur J Soc Sci.

[CR32] Odeyemi IA, Nixon J (2013). Assessing equity in health care through the national health insurance schemes of Nigeria and Ghana: a review-based comparative analysis. Int J Equity Health.

[CR33] (2013). OECD Outlook 2013 How Does Mexico Compare?”.

[CR34] Parrenas RS (2001). Servants of Globalization: Women.

[CR35] Patel V, Prince M (2001). Ageing and mental health in a developing country: who cares? Qualitative studies from Goa, India. Psychol Med.

[CR36] Prince M, Dementia Research G (2004). Care arrangements for people with dementia in developing countries. Int J Geriatr Psychiatry.

[CR37] Prince M, Ferri CP, Acosta D, Albanese E, Arizaga R, Dewey M, Gavrilova SI, Guerra M, Huang Y, Jacob KS, Krishnamoorthy ES, McKeigue P, Rodriguez JL, Salas A, Sosa AL, Sousa RM, Stewart R, Uwakwe R (2007). The protocols for the 10/66 dementia research group population-based research programme. BMC Public Health.

[CR38] Prince M, Acosta D, Albanese E, Arizaga R, Ferri CP, Guerra M, Huang Y, Jacob KS, Jimenez-Velazquez IZ, Rodriguez JL, Salas A, Sosa AL, Sousa R, Uwakwe R, van der Poel R, Williams J, Wortmann M (2008). Ageing and dementia in low and middle income countries-Using research to engage with public and policy makers. Int Rev Psychiatry.

[CR39] Rofman R, Oliveri ML (2012). Pension Coverage in Latin America.

[CR40] Scott J (2008). Redistributive constraints under high inequality: The case of Mexico, Centro de Investigación y Docencia Económicas (CIDE).

[CR41] Sen A (1987). Gender and Cooperative Conflicts. World Institute for Development Economics Research.

[CR42] Shaji KS, Smitha K, Lal KP, Prince MJ (2003). Caregivers of people with Alzheimer's disease: a qualitative study from the Indian 10/66 Dementia Research Network. International Journal of Geriatric Psychiatry.

[CR43] Sousa RM, Ferri CP, Acosta D, Albanese E, Guerra M, Huang Y, Jacob KS, Jotheeswaran AT, Rodriguez JJ, Pichardo GR, Rodriguez MC, Salas A, Sosa AL, Williams J, Zuniga T, Prince M (2009). Contribution of chronic diseases to disability in elderly people in countries with low and middle incomes: a 10/66 Dementia Research Group population-based survey. Lancet.

[CR44] Sousa RM, Ferri CP, Acosta D, Guerra M, Huang Y, Jacob K, Jotheeswaran A, Hernandez MA, Liu Z, Pichardo GR, Rodriguez JJ, Salas A, Sosa AL, Williams J, Zuniga T, Prince M (2010). The contribution of chronic diseases to the prevalence of dependence among older people in Latin America, China and India: a 10/66 Dementia Research Group population-based survey. BMC Geriatr.

[CR45] Tetreault Weber D, Valencia Lomelí E, Foust Rodríguez D (2012). Social protection systems in Latin America and the Caribbean: Mexico.

[CR46] Uwakwe R, Ibeh CC, Modebe AI, Bo E, Ezeama N, Njelita I, Prince MJ (2009). The Epidemiology of Dependence in Older People in Nigeria: Prevalence, Determinants, Informal Care, and Health Service Utilization. A 10/66 Dementia Research Group Cross‒Sectional Survey. Journal of the American Geriatrics Society.

[CR47] Varley A, Blasco M (2003). Older women’s Living Arrangements and Family Relationships in Urban Mexico.

[CR48] Vilela A (2013). Pension Coverage in China and the Expansion of the New Rural Social Pension. Briefings on Social Protection in.

[CR49] (2010). Measuring Health and Disability: Manual for WHO Disability Assessment Schedule (WHODAS 2.0)”.

[CR50] Yach D, Hawkes C, Gould CL, Hofman KJ (2004). The global burden of chronic diseases: overcoming impediments to prevention and control. Jama.

[CR51] Yang G (2009). New masters, New servants: migration, development and women workers in China. China Quarterly.

