# Supplementary material for: Exploring the economic and social effects of care dependence in later life: protocol for the 10/66 research group INDEP study
Source: Springerplus. 2014 Jul 28;3:379. doi: 10.1186/2193-1801-3-379 (PMC4124109; doi:10.1186/2193-1801-3-379)
Supplement: Supplementary file 1 — Additional file 1: Table S1: Pensions, Healthcare Insurance and Healthcare Financing in Mexico, Peru, China and Nigeria (International; Carranza, et al 2012; Tretreault et al 2012; Scott, 2008; Knaul et al., 2012; Knaul & Frenk, 2005; International; Lavigne 2013; Rofman & Oliveri, 2012; International; Alcalde-Rabanal, et al., 2011; Vilela, 2013; Li, et al., 2011; Liu, 2012; Adebayo & Dada, 2012; Dostal, 2010; Odeyemi & Nixon, 2013). (DOCX 28 KB) [file 40064_2014_1086_MOESM1_ESM.docx]

| **Supplementary Table 1. Pensions, Healthcare Insurance and Healthcare Financing in Mexico, Peru, China and Nigeria** | | | | | |
| --- | --- | --- | --- | --- | --- |
|  | **Social Pensions** | **Contributory Pensions** | **Pension Coverage** | **Healthcare Insurance** | **Healthcare Financing** |
| **Mexico** | **Pension Alimentaria Ciudadana (2003)**   - Universal in Mexico City for those >68yrs - 897 pesos (68USD)/m - 8.5% of average income, 273% international poverty line - 2 million recipients - Covers 19% population 60yrs and above   **70 y mas (2007)**   - Universal for residents of towns <30 000 inhabitants - 500 pesos / (38USD)/m - 4.8% average income - 152% of international poverty line - 1.9 million recipients - Covers 18% population 60yrs and above   (HelpAge International, 2013) | **Instituto Mexicano del Seguro Social (IMSS)**   - 6.3% of earnings - Plus federal government funded Social Contribution (progressive scheme) for those earning up to 15 x minimum wage ([Carranza, Melguizo, & Tuesta, 2012](#_ENREF_2)) - Open to domestic workers, workers from family companies and members of cooperatives but mostly workers from private companies (Tretreault Weber et al 2012)   **Other Contributory Pensions**   - State workers with contributions for >25yrs (ISSSTE) - Members of Mexican armed forces (ISSFAM) - Trade Union of Oil Workers (STPMR) (Tretreault Weber et al 2012) | 50% of older people not accessing social or contributory pensions ([Scott, 2008](#_ENREF_10)) | **Seguro Popular de Salud (SPS)**   - Comprehensive package of essential services - 2003 reforms: goal was to achieve universal coverage (transition period to 2012) - 50 million people previously excluded from public, social insurance - 98% of Mexicans registered with health insurance by end of 2011 ([Knaul et al., 2012](#_ENREF_5)) | - Less than half of total health expenditure is public ([Knaul & Frenk, 2005](#_ENREF_4)) - Funding of SPS is shared between federal and state government (and employer- if employed) - Fund for Protection against Catastrophic Health Expenditures (part of SPS) covers costly, specialised interventions but host of common, costly, treatable chronic diseases not covered ([Knaul, et al., 2012](#_ENREF_5)) |
| **Peru** | **Pension 65 (2011)**   - For those aged >65yrs and not receiving other pensions - 9.3% of average national income, 204% of international poverty line - Covers 5% of population 60yrs and above - 250 soles (90USD)/m   (HelpAge International, 2013) | **Sistema Nacional de Pensiones (SNP)**   - 13% of earnings for at least 20yrs- worker’s contribution   **Cedula Viva (the Living Decree)**   - For teachers that started working <1980, workers at state companies and magistrates   **Sistema Privado de Pensiones, (SPP)**   - Set up in response to deficit in public pension funds - Individual capitalisation regime - Voluntary participation (Lavigne 2013) | An estimated 25% of older people receive a pension ([Rofman & Oliveri, 2012](#_ENREF_9)) | **Integral Health Insurance (SIS)**   - Government provision of health services to the uninsured - 18% of the population- mostly rural and marginal urban areas - Ministry of Health operates particular hospitals that offer services regardless of insurance status   **Es Salud**   - Covers approximately 20% of the population: formal sector workers, retirees and their families - Provides health services in own health centres- mainly in urban areas   **Armed Forces Medical Services**   - For the military, police and their families - Approximately 2% of the population   **10-20% of population is totally excluded from health system**  ([Alcalde-Rabanal, Lazo-González, & Nigenda, 2011](#_ENREF_1)) | - SIS is almost entirely funded by central government - Ministry of Health facilities offer services for discretionary sliding scale fee which may be covered by SIS - EsSalud covers most health needs but insufficient supply and long waiting list means that many use private services - EsSalud is financed by employer contributions - Most high-cost, chronic problems handled by Es Salud- ie. AIDS, dialysis ([Alcalde-Rabanal, et al., 2011](#_ENREF_1)) |
| **China** | **New Rural Social Pension Scheme (NRSPS) (2009)**   - Born out of reforms and pilot schemes in 2009- combination of social and contributory - 89 million people covered - Basic pension of 55 yuan (8.8USD)/m is payable to people over 60yrs whose children participate in scheme - 55 yuan is 14% of average national income - May be supplemented with local government revenues - Subsidised by central government- percentage varies by region   **Urban Social Pension Scheme**  **(USPS) (2011)**   - For urban areas (in cities with sufficient funds) - Follows similar design to NRSPS - Basic pension scheme contributions for those aged >16yrs, not employed in formal sector, older people not receiving BOISE - 2.6 million people receiving USPS (2011)   ([Vilela, 2013](#_ENREF_11)) | **New Rural Social Pension Scheme (NRSPS)**   - Those aged>16yrs, not in education or enrolled in urban pension scheme are eligible for voluntary participation - Individual contributions of 100-500 yuan (equivalent to 1.28-6.24 USD/m) Local government provides partial matched contribution 4.8USD/year - Those aged >45yrs encouraged to make larger contributions to meet shortfall - Those who have contributed for >15yrs will be eligible for basic flat rate pension   **Basic Old-Age Insurance Scheme (BOISE)**   - Employment-based urban system - Covers 63% of urban residents; 45% of workers in eligible industries ([Vilela, 2013](#_ENREF_11)) | 60% of people over 60yrs receiving a pension (HelpAge International, 2013) | **New Rural Cooperative Medical Schemes (NRCMS) (2003)**   - Covers 833 million of the rural population - Voluntary   **Urban Employee Basic Medical Insurance (UEBMI) (1998)**   - 200 million participants - For urban residents with formal employment/retirees, public and private sector - Compulsory - Does not cover dependents - Outpatient & inpatient care- must obtain care from designated facilities ([Li, Yu, Butler, Yiengprugsawan, & Yu, 2011](#_ENREF_6))   **Urban Resident Basic Medical Insurance (URBMI) (2007)**   - 221 million residents; 16.5% of population (2011) - Voluntary, for urban residents without formal employment - Mainly covers inpatient and outpatient services for catastrophic illness- chronic conditions only covered in most affluent districts - Research suggest that URBMI does not reduce out-of-pocket spending (increased healthcare use, more use of higher-level providers) - Improved utilization by elderly, low and middle income residents ([Liu, 2012](#_ENREF_7)) | **NRCMS**   - Contributions from central & local government and individuals - 80RMB per individual from government plus 20 RMB from individual per yr - Average reimbursement is 36.4% of inpatient care costs   **UEBMI**   - 6% of employees salary contributed by employer; 2% contributed by employees - Reimbursement caps vary widely according to local financing levels, average is 66.2% for inpatient costs ([Li, et al., 2011](#_ENREF_6))   **URBMI**   - URBMI is financed by individual contributions (lower than UEBMI) and government subsidies shared between central and local governments - Subsidy amounts to 36% of cost for adults and 56% for children - Reimbursement caps vary widely according to local financing levels ([Liu, 2012](#_ENREF_7)) |
| **Nigeria** | **Ekiti State Social Security Scheme (2011)**   - For those aged >65yrs in Ekiti state only - 22.7% of average income, 135% of international poverty line - 20 000 people receiving this pension out of an estimated 120 000 people >65yrs in the state (Adebayo & Dada, 2012)   Osun State   - For 1602 older people identified as the “most vulnerable” - N10 000 (66USD)/M - 50% of the national average income - Data on number of older people in the state not available but larger than Ekiti (HelpAge International, 2013) | **National Pension Commission**   - 2004 reforms to reform and simplify various schemes (federal, state, local government, military, police, security services etc.) as well as formal private sector schemes - Privately managed, funded pension accounts- covering 4 million Nigerians - Limited to formal sector employees - Public and private sector workers: 7.5% of salary (matched by employer) - Armed Forces: 2.5% of salary; 12.5% contributed by government ([Dostal, 2010](#_ENREF_3)) | 4 million of an estimated workforce of 40 million (10%) enrolled in contributory pensions ([Dostal, 2010](#_ENREF_3)) | **National Health Insurance Scheme (2005)**   - Only 3% population enrolled (mainly federal government employees) - State governments expected to adopt programme for its employees and dependence but only enacted by two states (Cross River and Enugu) out of 34 - Consists of three programmes (see below)   **Formal Sector Social Health Insurance (FSHIP)**   - Employers enrol employees with a particular NHIS affiliated Health Maintenance Organisation   **Urban Self-Employed Social Health Insurance Programme (USSHIP)**   - Covers user groups with common economic activities (open to those that contain at least 500 members   **Rural Community Social Health Insurance Programme (RCSHIP)**   - Cohesive group of households/individuals to form a community ([Odeyemi & Nixon, 2013](#_ENREF_8)) | - 95% of healthcare costs paid by service-users - 1% of private expenditure attributed to private health insurance (<1million people) - 4.9% of total spending on healthcare is from donor funding - FSHIP- revenue raising shared: employer pays 10% of employees salary, employee pays 5% - USSHIP &RCSHIP- participants pay flat monthly rate depending on health package chosen by group ([Odeyemi & Nixon, 2013](#_ENREF_8)) |
